# Metformin Improves Autonomic Nervous System Imbalance and Metabolic Dysfunction in Monosodium L-Glutamate-Treated Rats

**DOI:** 10.3389/fendo.2021.660793

**Published:** 2021-06-04

**Authors:** Claudinéia Conationi da Silva Franco, Carina Previate, Amanda Bianchi Trombini, Rosiane Aparecida Miranda, Luiz Felipe Barella, Lucas Paulo Jacinto Saavedra, Júlio Cezar de Oliveira, Kelly Valério Prates, Laize Peron Tófolo, Tatiane Aparecida Ribeiro, Audrei Pavanello, Ananda Malta, Isabela Peixoto Martins, Veridiana Motta Moreira, Camila Cristina Ianoni Matiusso, Flávio Andrade Francisco, Vander Silva Alves, Ana Maria Praxedes de Moraes, Juliane Rocha de Sant Anna, Marialba Avezum Alves de Castro Prado, Rodrigo Mello Gomes, Elaine Vieira, Paulo Cezar de Freitas Mathias

**Affiliations:** ^1^ Laboratory of Secretion Cell Biology, Department of Biotechnology, Genetics and Cell Biology, State University of Maringá, Maringá, Brazil; ^2^ Carlos Chagas Filho Biophysics Institute, Federal University of Rio de Janeiro, Rio de Janeiro, Brazil; ^3^ Institute of Health Sciences, Federal University of Mato Grosso-Sinop, Sinop, Brazil; ^4^ Laboratory of Mutagenesis & Genetics, Department of Cell Biology and Genetics, State University of Maringá, Maringá, Brazil; ^5^ Department of Physiological Sciences, Federal University of Goiás, Goiânia, Brazil; ^6^ Postgraduate Program on Physical Education, University Católica of Brasília, Brasília, Brazil

**Keywords:** metformin, insulin secretion, acetylcholine, autonomic nervous system, MSG-obese rats

## Abstract

Metformin is an antidiabetic drug used for the treatment of diabetes and metabolic diseases. Imbalance in the autonomic nervous system (ANS) is associated with metabolic diseases. This study aimed to test whether metformin could improve ANS function in obese rats. Obesity was induced by neonatal treatment with monosodium L-glutamate (MSG). During 21–100 days of age, MSG-rats were treated with metformin 250 mg/kg body weight/day or saline solution. Rats were euthanized to evaluate biometric and biochemical parameters. ANS electrical activity was recorded and analyzed. Metformin normalized the hypervagal response in MSG-rats. Glucose-stimulated insulin secretion in isolated pancreatic islets increased in MSG-rats, while the cholinergic response decreased. Metformin treatment normalized the cholinergic response, which involved mostly the M3 muscarinic acetylcholine receptor (M3 mAChR) in pancreatic beta-cells. Protein expression of M3 mAChRs increased in MSG-obesity rats, while metformin treatment decreased the protein expression by 25%. In conclusion, chronic metformin treatment was effective in normalizing ANS activity and alleviating obesity in MSG-rats.

## Introduction

The incidence of non-communicable diseases such as diabetes, obesity, and hypertension has been growing in the last five decades, reaching high prevalence in developing countries, with serious consequences to the future of human beings (1, 2). These diseases manifest mostly as common metabolic dysfunctions such as glucose intolerance, insulin resistance, hyperglycemia, hyperinsulinemia, and dyslipidemia, among other clinical conditions (3). To treat 350 million people with type 2 diabetes worldwide, as estimated by the World Health Organization, private and public institutions follow treatment programs that use drugs to ameliorate disease symptoms. One of these antidiabetics drugs is metformin, which is used to treat diabetes worldwide since 1950 (4). Metformin essentially contributes to improve glucose metabolism, decreasing hyperglycemia of diabetic type 2 patients. Although, used as antidiabetic a long time, metformin action mechanism is not yet clarified. It must highlight that metformin induces decrease of glucose blood concentration by inhibiting hepatic glucose production *via* gluconeogenesis, and improving tissue insulin sensitivity, mostly white fat and muscle tissue (5). In addition, there is evidence that metformin improves hypertension and dyslipidemia (6–8).

Due to the effectiveness of metformin treatment, the drug has been prescribed after metabolic disease diagnosis. Cardiometabolic disease onset has been linked to metabolic dysfunction in the past (9). Unfortunately, clinicians cannot anticipate cardiometabolic disease diagnosis to limit the effects of overt diabetes, for example, by treating patients with metformin. Considering the developmental origin of health and disease (DOHaD) concept, at least a few of the causes contributing to the increase in cardiometabolic diseases can be attributed to stressful insults during perinatal life (10).

Several early clinical signals are strongly associated with diseases in adulthood. Low/high birth weight and early obesity (infancy and adolescence) indicate a high risk of developing obesity, type 2 diabetes, and hypertension, among other cardiometabolic diseases, later in life (6). There is evidence that the brain controls metabolism and that the autonomic nervous system (ANS) can directly regulate blood glucose levels. The parasympathetic branch stimulates pancreatic beta-cells to potentiate insulin secretion and enables enhanced tissue glucose uptake. In contrast, the sympathetic nervous system reduces insulin secretion, leading to an increase in blood glucose. Both humans and animals with metabolic dysfunctions exhibit ANS impairments (9, 10).

Monosodium L-glutamate (MSG) treatment during neonatal life produces cardiometabolic dysfunction in rodents. Rats and mice experience obesity, dyslipidemia, glucose intolerance, insulin resistance, hyperinsulinemia, hypertension, and ANS impairment, among other hallmarks, when they reach adulthood (7, 8). These conditions are associated with impaired pancreatic beta-cells function and alterations in cholinergic muscarinic receptor (mAChR) activities (11). In this study, we used MSG-treated rats as an animal model of obesity and aimed to study whether ANS imbalance is attenuated through early metformin treatment in rats.

## Materials and Methods

### Animals

During all experimental protocols, male Wistar rats were kept at a temperature of 23 ± 2°C, a light/dark cycle of 12 h, and had free access to water and a standard rodent chow diet (Nuvital^®^, Curitiba, PR, Brazil). Experiments were approved by the Ethics Committee for Animal Experimentation (CEUA) of the State University of Maringá.

To induce obesity, male offspring rats received subcutaneous injections of MSG [4 mg/kg body weight (bw); Sigma-Aldrich, Germany] in the cervical area during the first five days of life (12).

### Metformin Treatment

To evaluate the effect of chronic metformin treatment in the metabolism of MSG-rats, the animals were divided into two groups during weaning. The MSG-metformin group received metformin hydrochloride (Medley, Brazil) dissolved in water at a dose of 250 mg/kg of bw using a gavage once a day from 21 to 100 days of age. The MSG-water group (control) received water. Compared with other studies, the metformin dose used in the present work had no detectable toxic effects in the animals (13, 14). Twenty-four hours after the last gavage with metformin, treated and untreated animals were used in the subsequent experimental proceedings.

### Metabolic and Biometric Parameters

Under ketamine and xylazine anesthesia (3 and 0.6 mg/100 g bw, respectively), a silicone cannula was implanted into the right jugular vein of the animals from both groups and stabilized on the dorsal region of the neck. The cannula was previously treated with heparin diluted in saline solution [50 IU heparin/ml, 0.9% of saline solution] to avoid blood clots. Twenty-four hours after the surgery and after 12 h of fasting without anesthesia, a glucose load (1 g/kg bw) was infused into the animals’ vein through the cannula. Blood samples (350–400 µl) were collected immediately before the glucose load (time 0) and at 5, 15, 30, and 45 min after glucose administration.

To study the role of the mAChR on glycemic levels, 5 min before glucose infusion, an intraperitoneal injection of acetylcholine (ACh), a cholinergic physiological agonist (ACh, 27 nmol/l/kg bw), or atropine, a cholinergic non-selective antagonist (Atr; 20 nmol/l/kg bw), was administered to another batch of animals from both groups.

To verify whether the cholinergic response was related to M3 mAChR function, the effects of a selective M3 mAChR antagonist, 4-diphenylacetoxy-N-methylpiperidine methiodide (4DAMP, 0.21 µmol/l/kg bw), were assessed in both experimental groups. The untreated rats received saline solution (0.9% of NaCl). Plasma obtained from blood samples was stored at −20°C for subsequent determination of glycemia and insulinemia. Glucose concentrations were measured by the glucose oxidase method, using a commercial kit (Gold Analisa^®^, Belo Horizonte, MG, Brazil). The measurements were performed in a single assay.

### Pancreatic Islets Isolation

Islets were isolated by collagenase digestion of the pancreas, as previously described (15). At 101 days of age, rats were decapitated, and the abdominal wall was cut open. Then, 8 ml of Hanks buffered saline solution (HBSS), [(mmol l^−1^) (NaCl, 136.9; KCl, 5.4; MgSO_4_7H_2_O, 0.81; Na_2_HPO_4_, 0.34; KH_2_PO_4_, 0.44; CaCl_2_2H_2_O, 1.26; NaHCO_3_, 4.16; glucose, 0.06; BSA (bovine serum albumin) 15 and (O_2_, 95% + CO_2_, 5% mixed)/10 min, pH 7.4)] containing (collagenase type XI, 0.1% plus BSA, 5% and HEPES [N-(2-hydroxyethyl-piperazine)-N′-(2-ethanesulphonic acid)], 0.6%, Sigma-Aldrich^®^, St. Louis, MO, USA), was injected into the common bile duct. The pancreas swelled rapidly due to the collagenase solution and was excised and incubated in a glass beaker for 17–18 min at 37°C. The suspension was then discarded and washed with HBSS in three continuous washes. Islets were collected with the aid of a stereomicroscope. At least three rats from three different litters were used for each experimental procedure in each group.

### Insulin Secretion

Isolated islets were pre-incubated with baseline glucose concentration (5.6 mmol l**^−1^**) for 60 min in 1 ml of normal Krebs–Ringer solution [(mmol l**^−1^**): NaCl, 115; NaHCO_3_, 24; KCl, 1.6; MgCl6H_2_O, 1; CaCl_2_2H_2_O, 1; BSA, 15] at pH 7.4 containing 5.6 mmol l**^−1^** glucose. The Krebs–Ringer solution was gassed with (O_2_, 95% + CO_2_, 5% mixed) to maintain a pH of 7.4. To study the insulinotropic effect of glucose, the islets were incubated for an additional 60 min in Krebs–Ringer solution containing glucose at concentrations 5.6 and 8.3 mmol l**^−1^** after pre-incubation.

To study mAChR function, the islets were incubated for 60 min in Krebs–Ringer solution containing 8.3 mmol l**^−1^** glucose or 8.3 mmol l**^−1^** glucose plus 10 μmol l**^−1^** ACh in the presence of neostigmine (10 μmol l**^−1^**). Neostigmine is an inhibitor of acetylcholinesterase and avoids ACh degradation in the isolated pancreatic islets, ensuring a cholinergic effect.

Additionally, to study the function of the insulinostatic and insulinotropic mAChR subtypes, another batch of isolated islets was incubated. In addition to glucose (8.3 mmol l**^−1^**), ACh (10 μmol l**^−1^**), and neostigmine (10 μmol l**^−1^**), we used either Atr (Atropine,10 mmol l**^−1^**) or one of the selective antagonists for the M1 (pirenzepine-PZP: 100 mmol l**^−1^**), M2 (methoctramine MTT: 1 mmol l**^−1^**), and M3 (4-DAMP: 100 mmol l**^−1^**) mAChR subtypes.

To study the insulinotropic response of pancreatic islets to high cholinergic signal stimulation, another batch of isolated islets was incubated with glucose (8.3 mmol l**^−1^**), neostigmine (10 μmol l**^−1^**), and ACh at different concentrations: 1; 10; 100, and 1,000 μmol l**^−1^**. Insulin secretion was determined based on the concentration in the supernatant of the incubated solution and insulin levels were measured by radioimmunoassay (RIA) with a gamma counter (2470 Wizard2 Automatic Gamma Counter, PerkinElmer). Human insulin was used as a standard, and an anti-rat insulin antibody (Sigma-Aldrich) and ^125^I-labeled recombinant human insulin (PerkinElmer) were used in detection of insulin. The intraassay and inter assay coefficients of variation were 12.2 and 9.8%, respectively, for insulin. The limit of detection was 0.006 ng/ml. The measurements were performed in a single assay.

The drugs described above for studying MAChR function were purchased from Sigma-Aldrich (St. Louis, MO, USA).

### Parasympathetic and Sympathetic Activities

At 101 days of age and after a 12-hour fasting period, rats from each experimental group were anesthetized with thiopental (45 mg/kg bw) in a batch, and a longitudinal surgical incision was performed on the anterior cervical region. Under a dissection microscope, the left superior branch of the vagus nerve, close to the trachea, was isolated and placed over a silver electrode inside a Faraday cage to avoid any electromagnetic interference, as previously described (16).

After 12 min of vagus nerve electrical activity recordings, the sympathetic branch nerve of the superior cervical ganglia was dissected, and the electrode was placed under the sympathetic branch nerve. Firing rates were obtained as described for the vagus nerve.

### Western Blotting

The M3mAChR protein content in the isolated pancreatic islets from 101-day-old rats was determined by western blotting. In total, 300 islets from both experimental groups were frozen at −80°C and subjected to posterior sonication (Sonic Dismembrator Model 100, Thermo Fisher Scientific, Waltham, MA, USA) in lysis buffer (in mmol l-1: HEPES, 50; MgCl_2_, 1; and EDTA, 10; plus, Triton X, 1%, v/v) containing a protease inhibitor cocktail (Roche^®^). The islets were then centrifuged at 12,000 rpm for 5 min at 4°C. The total protein content was determined using a BCATM Protein Assay Kit (Thermo Scientific^®^, Rockford, IL, USA) and a microplate reader (Multi-Mode Reader, FlexStation^®^ 3 Benchtop, Molecular Devices, Sunnyvale, CA, USA). The samples were treated with Laemmli sample buffer (w/v: glycerol, 20%; β-mercaptoethanol, 10%; 10% sodium dodecyl sulfate (SDS), 40%; and 0.5 mol l^−1^ Tris, pH 6.8, 0.5%; plus, deionized water and bromophenol blue). Total protein extracts (40 µg) from the pancreatic islets were separated using 10% SDS-PAGE at 90 V for 120 min. The proteins were transferred from the gel to a nitrocellulose membrane using the TransBlot^®^ Semi-Dry Electrophoretic Transfer Cell (Bio-Rad^®^, Hercules, CA, USA) and blocked with 5% skim milk in Tween-Tris-buffered saline (TTBS; Tris–HCl, 1 mol l^−1^; NaCl, 5 mol l^−1^; and Tween 20, 0.05%, v/v) for 90 min under continuous shaking. The blotted membranes were incubated overnight at 4°C with rabbit anti-M3mAChR (Sigma-Aldrich^®^, St. Louis, MO, USA) polyclonal primary antibody at 1:1,000 dilution, followed by incubation with peroxidase-conjugated anti-rabbit antibodies at 1:5,000 dilution (Sigma-Aldrich^®^, St. Louis, MO, USA). The antibodies were diluted in buffer (20 mmol l^−1^ Tris–HCl, 137 mmol l^−1^ NaCl, and 0.05% Tween 20). Immunoreactive proteins were visualized with ECL (GE Healthcare, Buckingham, Shire, UK) and a scanner (Amersham StormTM 860 Imaging System, Gene Tool, Milpitas, CA, USA). The bands were quantified by densitometry using Image J 1.4 software (Wayne Rasband, National Institutes of Health, Bethesda, MA, USA). β-Actin protein content (Santa Cruz Biotechnology^®^, Santa Cruz, CA, USA; diluted 1:1,000 in TTBS) was utilized for normalization.

### Statistical Analyses

Data were analyzed with one-way analysis of variance (ANOVA), followed by Bonferroni test and Student’s t-test. Data are presented as mean ± SEM, and p values of less than 0.05 were considered statistically significant. Analyses were carried out using GraphPad Prism, version 6.0, for Windows (GraphPad Software, La Jolla CA, USA).

## Results

### Metabolic Profile

As shown in **Table 1**, chronic metformin treatment led to a 20% decrease in the bw of treated animals compared with that in untreated animals (p <0.0001). Metformin treatment did not affect the body length in any group. The Lee index was reduced by 6.7% in metformin-treated rats (p <0.0001) compared with that in untreated animals. The mesenteric and epididymal adipose tissue weights were higher in MSG-water controls, whereas metformin treatment reduced both fat pads (p <0.0001). As shown in **Table 1**, rats from the MSG-water control group had a 2.5-fold higher insulinemia than rats from the metformin-treated group (p <0.0001).

**Table 1:** Biometric parameters of the monosodium L-glutamate (MSG) rats treated or no with metformin.

Biometric Parameters	MSG-Water	MSG-Metformin
Final body weight (g)	294.1 ± 6.3	233.5 ± 8.4^****^
Body length (cm)	19.52 ± 0.2	19.43 ± 0.2^ns^
Lee index	34.13 ± 0.2	31.81 ± 0.3^****^
Mesenteric fat pad (g/100 g of bw)	2.27 ± 0.11	1.36 ± 0.12^***^
Epididymal fat pad (g/100 g of bw)	2.18 ± 0.11	1.41 ± 0.07^****^
Fasting glycemia (mg/dl)	88.84 ± 3.04	83.33 ± 3.19^ns^
Fasting insulinemia (ng/ml)	1.01 ± 0.05	0.37 ± 0.01^***^
HOMA-IR^1^	4.81 ± 0.29	1.77 ± 0.09^****^
HOMA-b^2^	437.4 ± 87.7	193.6 ± 53.4^*^

The data represent the mean ± SEM, n = 8 from three distinct litters of each experimental group. The symbols represent significant differences by Student’s t test, ^*^p <0.03, ^***^p <0.0003, ^****^p <0.0001, ns, not significant; ^1^Homeostasis model assessment of insulin resistance. ^2^Homeostasis Model Assessment of β-cell function, SEM, standard error.

The Homeostatic Model Assessment of Insulin Resistance (HOMA-IR) and Homeostasis Model Assessment of β-cell function (HOMA-β) in the MSG-metformin rats decreased (63 and 56%, respectively, p <0.05) when compared to that in the MSG-water rats. Chronic metformin treatment significantly reduced insulin levels (–63%, p <0.001), thereby preventing the development of insulin resistance.

### Effect of mAChR Antagonists *In Vivo* During the Intravenous Glucose Tolerance Test on Glucose Homeostasis

**Figure 1:**
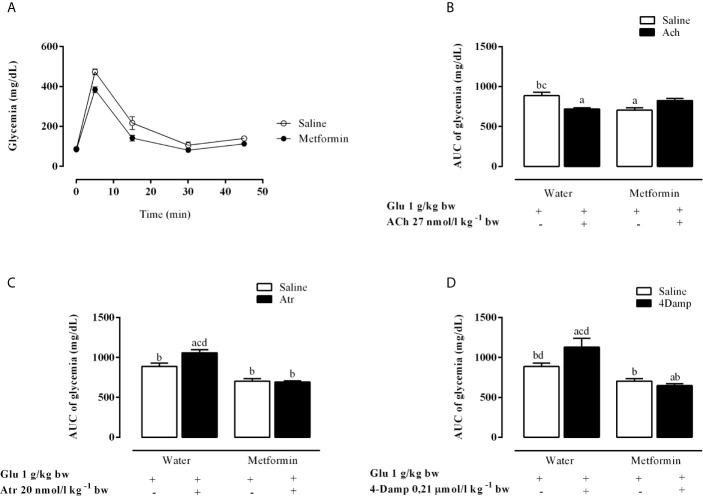
Effects of muscarinic receptor agonist and antagonists on glycaemia throughout the intravenous glucose tolerance test (ivGTT). **(A)** Effect of metformin treatment on glycaemia during the ivGTT. **(B–D)** show the glucose load-induced area under the glycaemia curve during IVGTT after pretreatment with Ach, Atr and 4-DAMP, respectively. The bars represent the mean ± SEM from 8–12 rats for each treatment, and letters over the bars represent the statistically significant differences using a one-way ANOVA (p <0.0003 and p <0.0001) among the groups.

As expected, chronic metformin treatment reduced hyperglycemia during intravenous glucose tolerance test (ivGTT), as shown in **Figure 1A**. **Figures 1B–D** show *in vivo* results of intraperitoneal administration of mAChR agonist and antagonist in metformin-treated and untreated animals. ACh caused a decrease of 18.8% in the glycemic area under the curve (AUC) values in the MSG-water rats during ivGTT, compared to animals that did not receive ACh (p <0.0003). The MSG-metformin rats presented a reduction of 14.6% in the glycemia AUC values compared to MSG-metformin-ACh (**Figure 1B**). Atr and 4-DAMP induced an increase of 19.5 and 27.3%, respectively, in the AUC of glycemia values of MSG-water animals. However, neither Atr nor 4-DAMP was able to increase glycemia in rats treated with metformin (**Figures 1C, D**).

**Figure 2:**
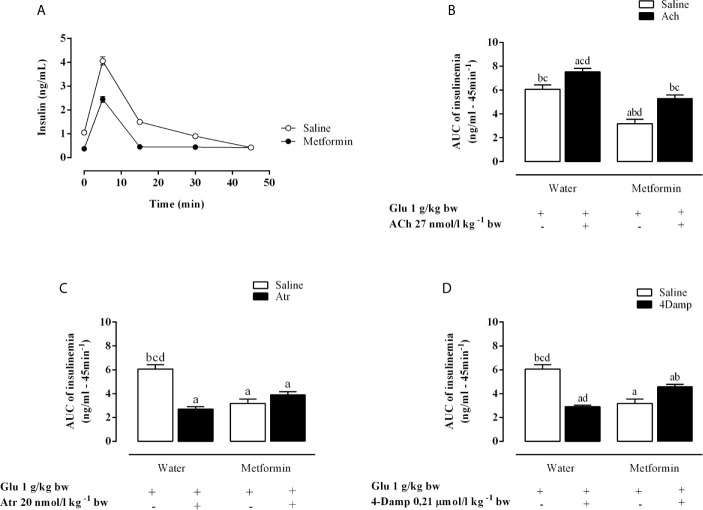
Effects of muscarinic receptor agonist and antagonists on insulinemia throughout the intravenous glucose tolerance test (ivGTT). **(A)** Effect of metformin treatment on insulinemia during the ivGTT. **(B–D)** show the area under the insulinemia curve during IVGTT induced by glucose load after pretreatment with Ach, Atr and 4-DAMP, respectively. The bars represent the mean ± SEM from 8–12 rats for each treatment, and letters over the bars represent the statistically significant differences using one-way ANOVA (p <0.0001) among the groups.

As shown in **Figure 2A**, chronic metformin treatment decreases insulin levels in the MSG-metformin rats. ACh increased insulinemia (AUC) by 24.2% during ivGTT in MSG-water rats (p <0.0001). Metformin treatment reduced this parameter, although MSG-metformin-ACh rats also displayed a 67.5% increase compared with that in MSG-metformin rats (**Figure 2B**). Both Atr and 4-DAMP reduced insulinemia AUC by 44.5% and 52.1%, respectively, in MSG-water controls (**Figures 2C, D**). Metformin treatment reduced insulin levels by 50%, and Atr and 4-DAMP did not alter the effect of metformin as shown by insulin AUC values (**Figures 2C, D**).

### Metformin Action on Glucose-Induced Insulin Secretion and Cholinergic Insulinotropic Effect in Isolated Pancreatic Islets

**Figure 3:**
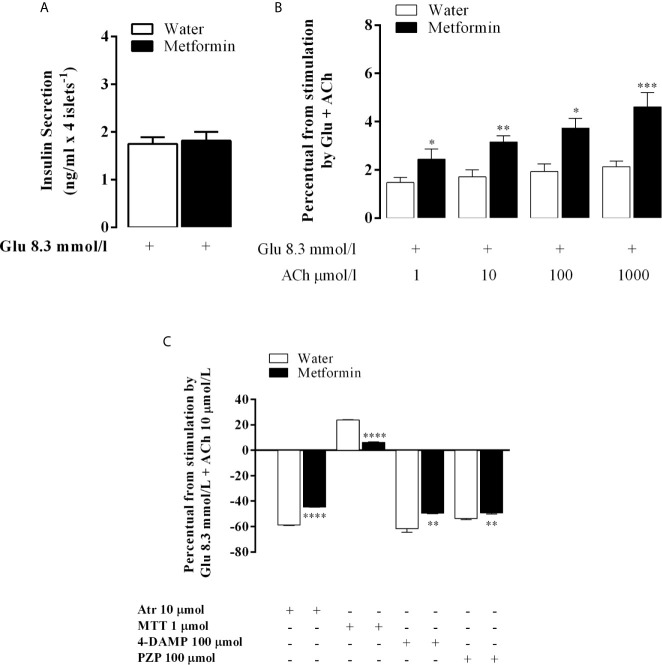
The *in vitro* effect of the muscarinic receptor agonist and antagonists on GIIS. Bars represent the mean ± SEM of insulin secretion from the pancreatic islets of eight rats that were obtained from four different litters. **(A)** Insulin secretion that was stimulated by 8.3 mmol l^−1^ Glu and potentiated by 10 mmol l^−1^ ACh. **(B)** The percentage of insulin release stimulated by 8.3 mmol l^−1^ Glu and 8.3 mmol l^−1^ Glu potentiated by 1, 10, 100 and 1,000 mmol l^−1^ ACh. **(C)** The bars above the 0 line representing (100% of 8.3 mmol 1^−1^ Glu-mediated insulin secretion throughout the 60 min of incubation in both groups islets) represent the percentage of the 10 μmol 1^−1^ Ach-mediated insulinotropic action. The line from 0 represents 100% of the 10 μmol 1^−1^ Ach-potentiated glucose-induced insulin release in both groups. The bars above or below the 0 line represent the agonist-provoked percentage increase or decrease [μmol/l: a nonselective antagonist (Atr, 10) or a selective M2mAChR (MTT, 1) or M3mAChR (4-DAMP, 100) antagonist) or (PZP, 100) in 10 μmol/l Ach-potentiated glucose-induced insulin release in both groups. *p<0.01, **p<0.002, ***p<0.0005, ****p<0.0001 indicated a significant difference between groups based on Student’s t-test.

As expected, chronic metformin treatment did not change glucose-induced insulin secretion (GIIS) (**Figure 3A**). ACh at different concentrations did not affect GIIS in the islets of MSG-water rats. However, it increased in a dose-dependent manner in MSG-metformin rats, as shown in **Figure 3B**.

Atr inhibited the ACh insulinotropic effect on GIIS in the islets of both groups. The decrease in insulin secretion was 58.8% in MSG-water and in 44.5% in MSG-metformin rats (p <0.0001). Likewise, 4-DAMP and PZP decreased insulin secretion by 61.5 and 53.7% (p <0.0001) in MSG-water rats, respectively, and by 49.5 and 49.3% in MSG-metformin rats, respectively (p <0.002). In contrast, MTT enhanced the insulinotropic effect of ACh by 24.0% in the islets from MSG-water rats and induced a 7% enhancement of the potentiating effect of ACh in the islets from MSG-metformin rats (**Figure 3C**, p <0.0001).

### Effect of Chronic Metformin Treatment on the Autonomous Nervous System Electrical Activity in MSG-Obese Rats

**Figure 4:**
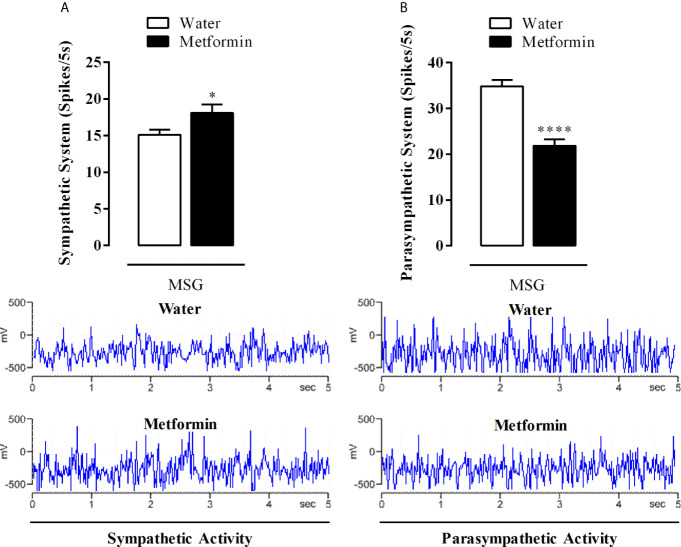
**(A)** Sympathetic and **(B)** parasympathetic electrical activity from the cervical superior nerves. The bars represent the mean ± SEM of the firing rates from sympathetic nerves and vagus nerves from 12 rats that were obtained from four different litters for each experimental group. Representative records of nerve discharges for each experimental group are in the lower panels. *p <0.02 and ****p <0.0001 by Student’s t-test.

While metformin treatment induced a 20% increase in the electrical activity of the sympathetic nerve in MSG-metformin rats (**Figure 4A**, p <0.02), the superior vagus nerve from MSG-metformin animals showed an activity reduction of 38% when compared with MSG-water animals (p <0.0001, **Figure 4B**).

### Effect of Metformin Treatment in the Protein Expression of Cholinergic Muscarinic Acetylcholine Receptors Subtypes in the Pancreatic Islets of MSG-Rats

**Figure 5:**
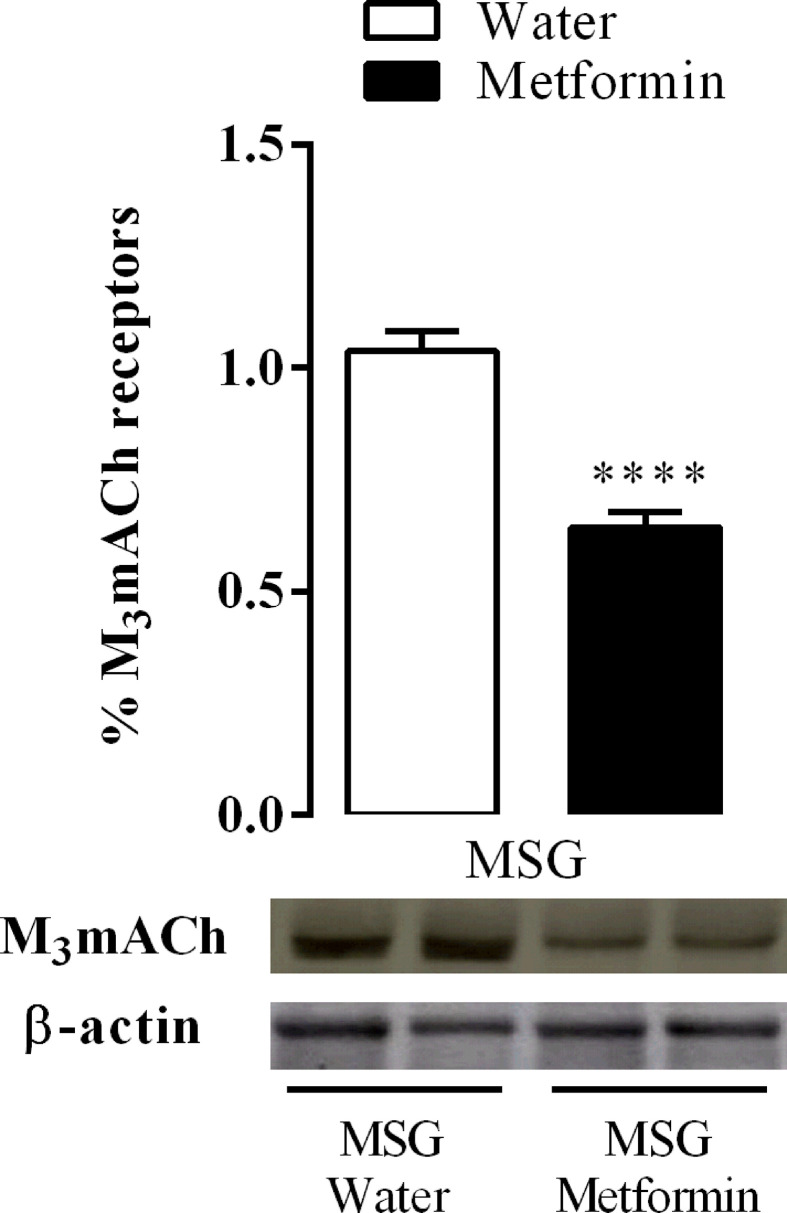
Effect of metformin treatment on M3AChR protein expression in the pancreatic islets from nine rats of three different litters per group for each experimental group. ****p <0.0001 by Student’s t-test. Representative western blotting images were originated from the same membrane.

**Figure 5** shows that chronic treatment with metformin decreased the protein expression of the M3 subtype cholinergic muscarinic acetylcholine receptor (mAChR) by 38% in the isolated pancreatic islets of the MSG-metformin group compared with that of the MSG-water group (p <0.0001).

## Discussion

The central finding of the current work is that early chronic metformin treatment improves the ANS activity of MSG-obese rats with metabolic dysfunction. However, our results do not explain whether metformin directly affects the ANS or if the improvement of metabolic dysfunction indirectly normalizes the ANS activity. There is evidence that metformin can cross the blood–brain barrier and localize in several brain areas (17). The hypothalamus is a metformin target and the ventromedial nucleus is stimulated leading to reduction of appetite (18–21).

As with other animal models with metabolic dysfunction, MSG-rats have high parasympathetic and low sympathetic activity. However, it has been reported that bilateral subdiaphragmatic vagotomy can improve metabolism malfunction (22, 23). The present study shows that superior vagal electrical activity decreases and sympathetic activity increases upon metformin treatment, leading to improvement of the ANS imbalance. Recently, our group showed that hyper vagal activity is an important underlying cause of obesity in MSG-rats (11). In addition, using agonists and antagonists of parasympathetic neurotransmitters, our work demonstrates that insulin secretion control improved in MSG-rats treated with metformin. Despite the limitations of the ivGTT technique, it can be seen that glucose intolerance and hyperinsulinemia are ameliorated in MSG-rats treated with metformin. It also shows that the animals respond to the exogenous ACh and the non-selective cholinergic blocker atropine. Many hormones, including enteric ones and neurotransmitters, are involved in regulating blood glucose levels *in vivo*. However, as a net response, exogenous ACh increases the insulin level and atropine reduces insulin levels and clearly influences glycemia before glucose bolus administration. Metformin-treated MSG-animals responded to ACh better than untreated MSG-rats. It is challenging to isolate factors that directly and indirectly influence insulin and glucose blood levels in a post-prandial situation. However, it has been shown that, at the same time, catecholamine and ACh inhibit and potentiate insulin secretion, respectively, after glucose infusion in animals and humans (24). Using an M3 mAChR non-selective antagonist, insulin levels decreased, showing that mAChRs are involved in parasympathetic signaling during the post-prandial phase emulating the effect of glucose bolus. Isolated pancreatic islets from MSG-rats treated with metformin rescue the cholinergic response in an M3 mAChR-dependent manner, but other mAChRs such as the M2 subtype mAChR are also involved in the process. Recently, our laboratory showed that mAChRs are involved in disrupting the cholinergic response to insulin release in MSG-rats (11).

The current study unequivocally shows that metformin treatment improves the cholinergic transduction associated with decreasing the expression of M3 mAChR. It has been shown that M3 mAChR function reduces vagal activity and that M3 mAChR expression is lower in MSG-rats (11). Interestingly, MSG-rats treated with metformin improved peripheral tissue insulin sensitivity, although it did not change glucose-stimulated insulin secretion in the isolated pancreatic islets (25). Our data suggest that insulin level control due to metformin treatment, including hormone releasing, improved independently of glucose stimulation. In line with our findings, recent data showed that islets isolated from MSG-rats treated with metformin responded to cholinergic signaling in a dose-dependent manner, unlike untreated rats that do not respond at all. In addition, *in vivo* assays showed increased insulin blood levels upon ACh stimulation. These findings suggest a relationship between decreased M3 mAChR protein expression and the cholinergic response recovered upon metformin treatment in pancreatic islets isolated from MSG-rats. Previous data showed that acute metformin treatment stimulated the release of glucagon-like peptide 1 in a cholinergic activity-dependent manner in rats (26). For the first time, the current study shows that metformin affects the ANS; however, the current study has some limitations, one of them is the animal house temperature. All the records regarding ANS electrical activities were done at 23 ± 2°C. It has been shown that thermoneutral conditions, it means 28°C, prevent interference to recording sympathetic activity. Low temperature induces “per se” enhancement of sympathetic tonus. To guarantee our results always we recorded ANS activity in MSG-obese rats that were not treated with metformin (27, 28). It is possible to improve metabolic dysfunction with antidiabetic drugs such as metformin, which targets the ANS. Approved drugs such as metformin can be used to develop new therapeutic strategies such as combining different drugs that normalize the ANS imbalance observed in cardiometabolic diseases.

## Data Availability Statement

The original contributions presented in the study are included in the article/supplementary material. Further inquiries can be directed to the corresponding author.

## Ethics Statement

The animal study was reviewed and approved by the Ethics Committee for Animal Experimentation of the State University of Maringá.

## Author Contributions

All authors contributed equally to the work. All authors contributed to the article and approved the submitted version.

## Funding

This work was supported by the Brazilian Federal Foundation, the Conselho Nacional de Desenvolvimento Científico e Tecnológico (CNPq), the Coordenação de Aperfeiçoamento de Pessoal de Nível Superior (CAPES), and the Paraná Science Foundation (Fundação Araucária) and Science without Borders Program (Programa Ciencias Sem Fronteiras). This study was funded by grant number 475765/2013-3 (CNPq) and AUX-PE-PNPD 2396/2009 (CAPES).

## Conflict of Interest

The authors declare that the research was conducted in the absence of any commercial or financial relationships that could be construed as a potential conflict of interest.

## Abbreviations

ACh, acetylcholine; ANS, Autonomic Nervous System; Atr, atropine; AUC, area under the curve; BSA, bovine serum albumin; bw, body weight; CEUA, Ethics Committee of the State University of Maringa; 4DAMP, 4-diphenylacetoxy-N-methylpiperidine methiodide; DOHaD, Developmental Origins of Health and Diseases; GIIS, glucose-induced insulin secretion; HBSS, Hanks buffered saline solution; HEPES, N-(2-hydroxyethyl-piperazine)-N’-(2-ethanesulphonic acid); HOMA-b, Homeostasis Model Assessment of β-cell function; HOMA-IR, Homeostasis model assessment of insulin resistance; ivGTT, intravenous glucose tolerance test; mAChR, cholinergic muscarinic acetylcholine receptor; MSG, Monosodium L-glutamate; MTT, methoctramine; PZP, pirenzepine; RIA, radioimmunoassay; SEM, standard error.
